# Geometric and Dosimetric Evaluation of the Automatic Delineation of Organs at Risk (OARs) in Non-Small-Cell Lung Cancer Radiotherapy Based on a Modified DenseNet Deep Learning Network

**DOI:** 10.3389/fonc.2022.861857

**Published:** 2022-03-15

**Authors:** Fuli Zhang, Qiusheng Wang, Anning Yang, Na Lu, Huayong Jiang, Diandian Chen, Yanjun Yu, Yadi Wang

**Affiliations:** ^1^ Radiation Oncology Department, The Seventh Medical Center of Chinese People’s Liberation Army (PLA) General Hospital, Beijing, China; ^2^ School of Automation Science and Electrical Engineering, Beihang University, Beijing, China

**Keywords:** non-small-cell lung cancer, organs at risk, medical image segmentation, deep learning, DenseNet, feature reuse

## Abstract

**Purpose:**

To introduce an end-to-end automatic segmentation model for organs at risk (OARs) in thoracic CT images based on modified DenseNet, and reduce the workload of radiation oncologists.

**Materials and Methods:**

The computed tomography (CT) images of 36 lung cancer patients were included in this study, of which 27 patients’ images were randomly selected as the training set, 9 patients’ as the testing set. The validation set was generated by cross validation and 6 patients’ images were randomly selected from the training set during each epoch as the validation set. The autosegmentation task of the left and right lungs, spinal cord, heart, trachea and esophagus was implemented, and the whole training time was approximately 5 hours. Geometric evaluation metrics including the Dice similarity coefficient (DSC), 95% Hausdorff distance (HD95) and average surface distance (ASD), were used to assess the autosegmentation performance of OARs based on the proposed model and were compared with those based on U-Net as benchmarks. Then, two sets of treatment plans were optimized based on the manually contoured targets and OARs (Plan1), as well as the manually contours targets and the automatically contoured OARs (Plan2). Dosimetric parameters, including Dmax, Dmean and Vx, of OARs were obtained and compared.

**Results:**

The DSC, HD95 and ASD of the proposed model were better than those of U-Net. The differences in the DSC of the spinal cord and esophagus, differences in the HD95 of the spinal cord, heart, trachea and esophagus, as well as differences in the ASD of the spinal cord were statistically significant between the two models (*P*<0.05). The differences in the dose-volume parameters of the two sets of plans were not statistically significant (*P*>0.05). Moreover, compared with manual segmentation, autosegmentation significantly reduced the contouring time by nearly 40.7% (*P*<0.05).

**Conclusions:**

The bilateral lungs, spinal cord, heart and trachea could be accurately delineated using the proposed model in this study; however, the automatic segmentation effect of the esophagus must still be further improved. The concept of feature map reuse provides a new idea for automatic medical image segmentation.

## Introduction

In China, lung cancer ranks first in both incidence and mortality rates, accounting for 17.9% of all new cases and 23.8% of total cancer deaths according to GLOBOCAN 2020 (1). Non-small-cell lung cancer (NSCLC) constitutes the majority of lung cancers. Radiotherapy (RT) is usually used in all stages of NSCLC treatment and is required at least once in more than half of patients for either cure or palliation. In a typical clinical workflow of RT, a radiation oncologist manually segments the tumor target and organs at risk (OARs) based on the information provided by CT, MRI and/or PET/CT images (2, 3). This process is often time consuming and laborious, and the quality of the segmentations largely depends on the experience of radiation oncologists. It is easy to distinguish the organs with high contrast on CT images; however, it is difficult to distinguish the boundary between tumor tissue and surrounding normal tissue with similar contrast. Moreover, inconsistencies in target and OARs segmentations have been reported for both inter-and intraobserver segmentation variability (4–8). These factors will affect the accuracy and efficacy of RT. Therefore, improving the consistency and efficiency of image segmentation becomes an urgent task.

In recent years, automatic medical image segmentation based on deep learning has become a popular research topic in RT, and several convolutional neural networks (CNNs) including U-Net, ResNet and DenseNet, have shown great success in autosegmentation of the target and OARs (9–16). DenseNet was proposed by Huang G et al. (17) in 2017, using the concept of feature map reuse to address the small training datasets in supervised learning. Moreover, DenseNet connects multiple dense blocks with a transition layer and concatenates the channels of each dense block feature map in series to increase the number of feature maps and improve the utilization rate of feature maps. Tong N et al. (18) improved the performance of their previous shape constrained fully CNNs for head and neck OARs segmentation on CT and low field MRI by incorporating generative adversarial network (GAN) and DenseNet. With the novel segmentation method, they showed that the low field MR images acquired on a MR guided radiation radiotherapy system can support accurate and fully automated segmentation of both bony and soft tissue OARs for adaptive radiotherapy. Fu J et al. (19) proposed a novel three-dimensional (3D) multipath DenseNet for generating the accurate glioblastoma (GBM) tumor contour from four multimodal pre-operative MR images. The multipath DenseNet demonstrated an improved accuracy over comparable algorithms in the clinical task of GBM tumor segmentation. To our best knowledge, there has not been an automatic segmentation study based on the DenseNet for NSCLC radiotherapy.

In this study, a deep learning model based on DenseNet and FCN (fully convolutional network) is proposed. The model uses the idea of feature reuse. It learns the planar distribution characteristics of OARs in CT images through a denseblock module and supplements details through long connections to achieve an end-to-end accurate OAR delineation for NSCLC patients.

## Materials and Methods

### Data Acquisition and Preprocessing

**Figure 1:**
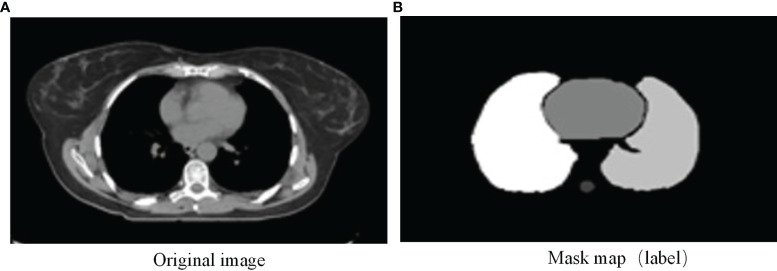
**(A)** Original image and **(B)** mask map (label).

The CT images of 36 NSCLC patients of the Seventh Medical Center of the PLA General Hospital were provided. The CT images were scanned on a Philips Brilliance Big Bore simulator (Philips Medical Systems, Madison, WI, USA) from the level of the larynx to the bottom of the lungs with a 3-mm slice thickness on helical scan mode. The study was approved by the Ethics Committee of the Seventh Medical Center of Chinese PLA General Hospital. All of the patients provided written consent for the storage of their medical information in the hospital database. Patients characteristics are shown in **Table 1**. By analyzing the DICOM file, the grayscale value of the original CT image was mapped to the range of 0-255, the window width was set to 400, and the window level was set to 40. Different manual OARs serving as the ground truth were filled with different grayscale values to generate mask images as training labels, as shown in **Figure 1**.

**Table 1:** Characteristics of patients in the training and testing sets.

Characteristics	Training set	Testing set
No. patients	27	9
Tumor site, right:left	16:11	3:6
Lobe location		
Upper left	7	5
Lower left	4	1
Upper right	7	1
Middle right	5	1
Lower right	3	1

The training dataset included 3803 CT images of 27 patients. The testing set included 567 images of 9 patients. In order to improve the utilization of the data and obtain a more stable model, the validation set was generated by cross validation and 6 patients’ images were randomly selected from the training set during each epoch as the validation set. After data cleaning and augmentation, these images were sent to the proposed model. The deep learning inference platforms used Tensorflow-gpu 1.7.0 as the underlying framework, Keras2.2.4 neural network library and python (version 3.6). All training, validating and testing were run on an NVIDIA GeForce GTX 1070 Ti GPU with 8 GB video memory. The starting and ending times of the manual and autosegmentation operations for each patient in the testing set were recorded.

### The Proposed Model for Segmentation

**Figure 2:**
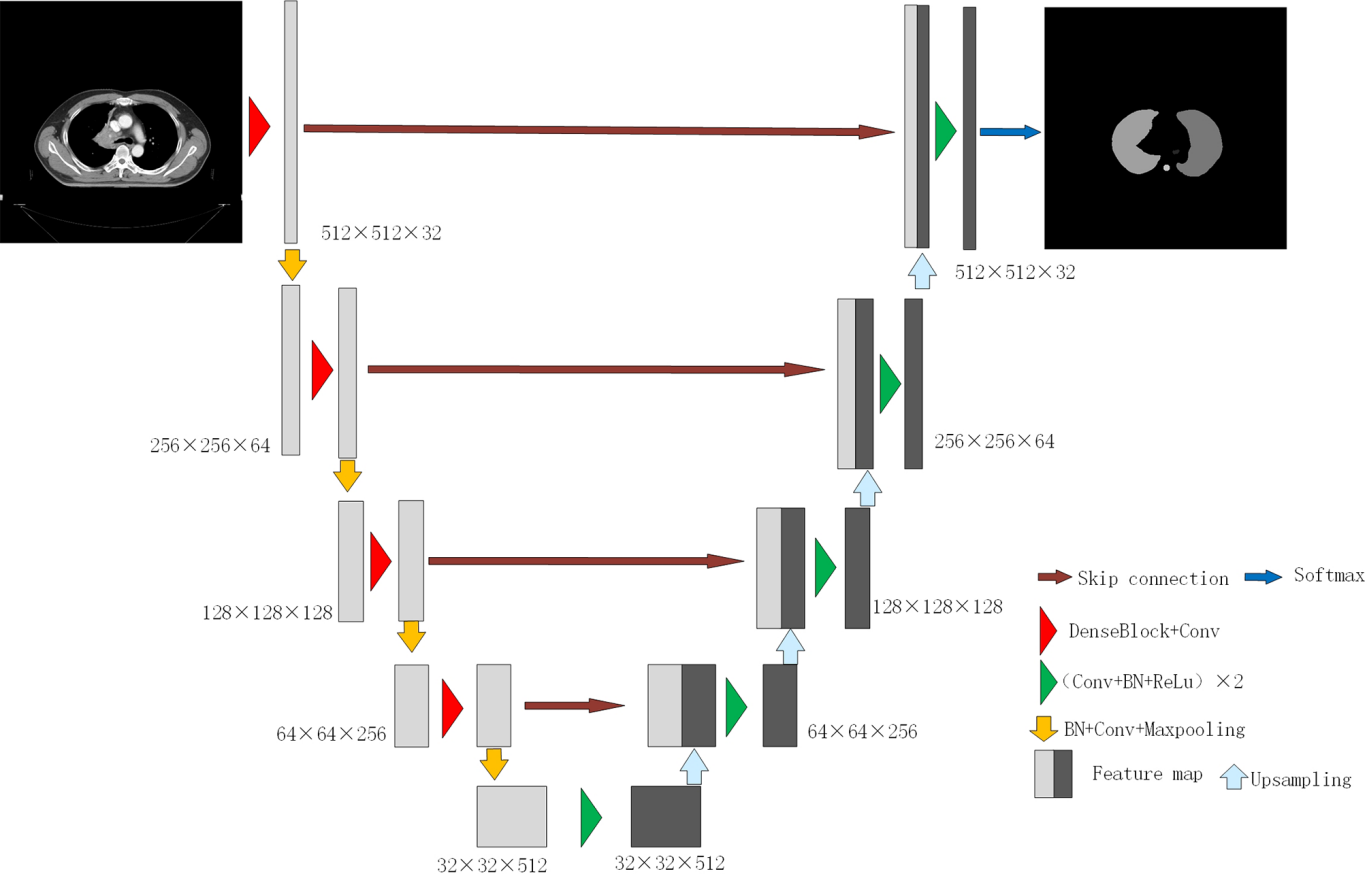
The architecture of the proposed model.

In this study, the model was trained to realize the autosegmentation of six OARs for NSCLC. The specific architecture of the model is shown in **Figure 2**. The segmentation process was mainly divided into two parts: the left half was called the analysis path, composed of a dense block module and a transition down module and connected by a short cut layer to extract image features; the right half was called the synthesis path, upsampled by a transition up transposition convolution module to recover the size of the feature map layer. To improve the accuracy of the reconstructed image and accelerate the convergence process of the network parameters, the feature maps of the same size in the analysis path were connected in series as the input of the next layer of the dense block.

**Figure 3:**
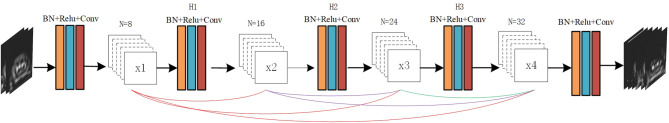
Scheme of dense block.

The input of each layer of the dense block was intensively composed of all of the outputs of its front layer after dense connection (as shown in **Figure 3**). The output of each layer had the following corresponding functional relationship with the output of other front layers:


(1) $$X_{i+1}=H({\text{X}}_{1},X_{2},\ldots ,X_{i})$$

where *H*(*) is a nonlinear function denoting a series of operations, including batch normalization (BN), ReLU activation, pooling and convolution, which are used to extract features, adjust the size of the feature map and reduce the channel dimension. The bottleneck architecture was set in each network since the operation of dense connections could bring about a surge in the number of channels and increase the difficulty of training. The bottleneck architecture used a 1×1 convolution kernel to realize cross-channel feature fusion and enhance the feature extraction ability of the network.

### Training of The Proposed Model

After cleaning and augmentation, data were sent to the model for training. The weight and bias of the network were updated using the cross entropy loss function as follows.


(2) $$L_{s}=-\sum_{i=1}^{k}\left(\text{y}\log\hat{y}+(1-\text{y})\log(1-\hat{y})\right)$$

(3) $$\hat{y}={\left(1+e^{w^{T}x+b}\right)}^{-1}$$

where *x* is the input of the network, $\hat{\text{y}}$ is the posterior probability output after network regression and *k* is the number of categories.

In this study, the early stopping module was added to detect the network accuracy and loss function value with the increase in the number of iterative epochs, and the network architecture based on DensNet56 in the 30th epoch was selected. During the network training process, the initial learning rate was set as 1e-3 and decreased with increasing epochs. This process ensured that the network could converge quickly in the initial stage of training, on the one hand, and avoided the problem of poor feature generalization due to network overfitting, on the other hand. In order to prevent the performance of the network from swinging at the local optimum, the Adam optimizer was used for training error. The Adam optimizer introduced the concept of second-order momentum, and the network weight was updated as the learning rate multiplied by the ratio of the gradient average to the square root of the gradient variance. The advantage of the method was that gradient updating was not only affected by the current gradient; but also by the accumulated gradient updating (20). The average segmentation time for the training set is approximately 12.58 min/epoch, the average segmentation time for a single 512×512 CT image is approximately 0.17 s, and the time for delineating all CT images of a patient is approximately 13.4 s.

### Accuracy Evaluation

#### Geometric Metrics

In this study, geometric evaluation metrics, including the Dice similarity coefficient (DSC), 95% Hausdorff distance (HD95) and average surface distance (ASD) (21), were used to assess the autosegmentation results of OARs based on the proposed model and were compared with those based on U-Net as benchmarks.

#### Dosimetric Metrics

To assess the dosimetric impact of the proposed model on treatment planning, we designed two sets of stereotactic body radiation therapy (SBRT) treatment plans for each patient in the testing set using manually segmented target volumes and OARs (Plan1), as well as the manually segmented target volumes and automatically segmented OARs (Plan2). Intensity modulated radiotherapy (IMRT) treatment plans were optimized with 6-MV photons using 5 coplanar beams. All of the plans were prescribed 6 Gy per fraction for 10 fractions and normalized as 100% prescription dose to 95% of the planning target volume (PTV). Dosimetric parameters including Dmax (meaning the dose received by 2% of the volume), Dmean, V40, V30, V20, V10, and V5 (meaning the volume receiving more than x Gy dose as a percentage of the total volume), were obtained and compared to assess the clinical feasibility of the proposed model. The dosimetric characteristics of OARs were gauged by the conformity index (CI) and homogeneity index (HI) of the PTV, so the CI and HI of the PTV were also calculated according to the formula in reference (22).

### Statistical Analysis

SPSS statistical software (version 20.0, SPSS Inc., Chicago, IL, USA) was used for statistical analysis. Wilcoxon’s signed rank test was used to compare the differences in DSC, HD95, ASD, and dosimetric parameters. Quantitative data are expressed as the mean ± standard deviation $\left(\bar{x}\pm s\right)$ , a value of *P*< 0.05 was considered statistically significant.

## Results

### Geometric Metrics

**Figure 4:**
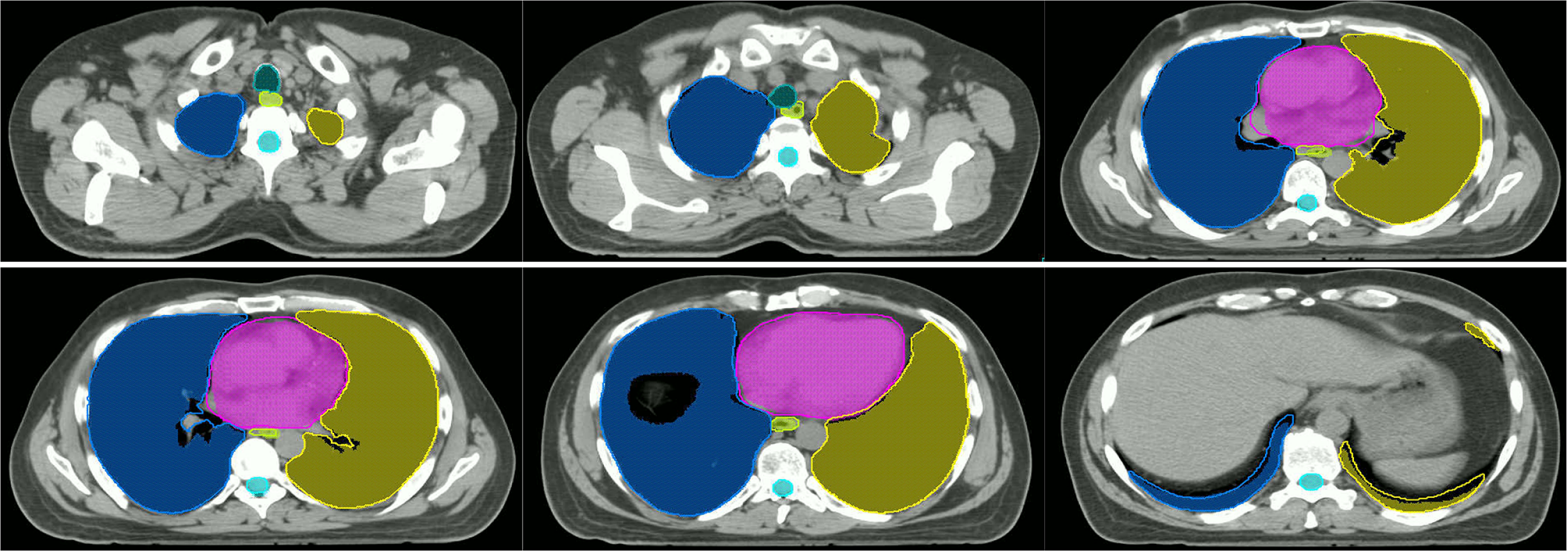
Comparison of manual and automatic segmentation of the OARs based on the proposed model (Color wash: the manual segmentation contour; line: the automatic segmentation contour).

The DSC, HD95, and ASD of OARs based on the proposed model and U-Net are listed in **Tables 2**–**4**, respectively. The proposed model showed better performance than U-Net, although there was no significant difference between the two models in several OARs (*P*>0.05). The comparison of the results between manual and automatic segmentation based on the proposed model for a typical patient is shown in **Figure 4**.

**Table 2:** Comparison of DSC of two models $\left(\bar{x}\pm s\right)$ .

	Spinal cord	Heart	Right lung	Left lung	Trachea	Esophagus
U-Net	0.82 ± 0.04	0.83 ± 0.09	0.96 ± 0.02	0.94 ± 0.02	0.86 ± 0.07	0.55 ± 0.11
Proposed	0.89 ± 0.01	0.86 ± 0.09	0.96 ± 0.01	0.95 ± 0.02	0.91 ± 0.03	0.67 ± 0.12
P value	0.008	0.535	0.897	0.709	0.212	0.008

**Table 3:** Comparison of HD95 (mm) of two models $\left(\bar{x}\pm s\right)$ .

	Spinal cord	Heart	Right lung	Left lung	Trachea	Esophagus
U-Net	3.75 ± 1.23	14.42 ± 2.94	7.24 ± 4.22	9.04 ± 5.97	4.46 ± 2.61	12.40 ± 5.99
Proposed	2.05 ± 0.38	9.75 ± 2.34	6.09 ± 1.56	6.47 ± 3.27	2.44 ± 1.17	6.14 ± 3.07
*P* value	0.000	0.008	0.897	0.260	0.039	0.008

**Table 4:** Comparison of ASD (mm) of two models $\left(\bar{x}\pm s\right)$ .

	Spinal cord	Heart	Right lung	Left lung	Trachea	Esophagus
U-Net	2.01 ± 0.70	7.70 ± 6.10	1.32 ± 0.45	1.57 ± 0.65	1.42 ± 0.87	6.95 ± 7.30
Proposed	0.81 ± 0.18	5.93 ± 4.03	1.11 ± 0.31	1.23 ± 0.54	0.94 ± 0.51	3.27 ± 2.67
*P* value	0.000	0.425	0.375	0.264	0.281	0.123

### Dosimetric Metrics

The dose-volume parameters of the OARs based on manual and automatic segmentation are listed in **Table 5**. There were no statistically significant differences between the dosimetric parameters of manual and automatically delineated OARs (*P*>0.05). The CIs of PTV in Plan1 and Plan2 were 0.74 ± 0.07 and 0.73 ± 0.07, respectively, while the HIs of PTV in Plan2 were 0.10 ± 0.02 and 0.09 ± 0.02, respectively. The differences in both CI and HI were not statistically significant (*P*>0.05).

**Table 5:** Dosimetric comparison of the PTV and OARs between manual and automatic segmentation-based plans $\left(\bar{x}\pm s\right)$ .

Dosimetric parameters		Plan1	Plan2	*P* value
PTV	CI	0.74 ± 0.07	0.73 ± 0.07	0.859
	HI	0.10 ± 0.02	0.09 ± 0.02	0.139
Spinalcord	Dmax (Gy)	18.66 ± 7.95	18.71 ± 7.36	0.678
Heart	V30 (%)	0.42 ± 1.16	0.43 ± 1.19	0.655
	V40 (%)	0.22 ± 0.63	0.22 ± 0.67	0.655
	Dmean (Gy)	1.71 ± 1.58	1.61 ± 1.53	0.441
Lung All	V5 (%)	27.55 ± 6.81	28.19 ± 6.78	0.515
	V10 (%)	15.49 ± 4.41	14.83 ± 4.54	0.953
	V20 (%)	9.40 ± 3.69	9.44 ± 3.89	0.859
	V30 (%)	6.57 ± 3.25	6.64 ± 3.36	0.263
	Mean (Gy)	6.49 ± 1.94	6.56 ± 2.01	0.173
Lung_L	V5 (%)	31.34 ± 14.34	30.80 ± 13.94	0.260
	V10 (%)	17.41 ± 17.79	17.16 ± 17.71	0.310
	V20 (%)	12.64 ± 14.34	12.48 ± 14.38	0.225
	V30 (%)	9.82 ± 11.68	9.69 ± 11.56	0.173
	Dmean (Gy)	8.07 ± 6.65	7.99 ± 6.65	0.477
Lung_R	V5 (%)	24.39 ± 8.38	24.48 ± 8.59	0.678
	V10 (%)	12.78 ± 11.37	12.89 ± 11.48	0.314
	V20 (%)	6.64 ± 8.31	6.69 ± 8.44	0.686
	V30 (%)	4.02 ± 5.28	4.07 ± 5.44	0.715
	Dmean (Gy)	5.14 ± 3.16	5.14 ± 3.29	0.477
Trachea	Dmean	6.00 ± 3.74	5.59 ± 3.47	0.139

### Delineating Time Analysis

The average time for manual segmentation by experienced radiation oncologists for 9 patients in the testing set was 15.2 min, while the total autosegmentation time of the 9 patients in the testing set was 9.0 min. Autosegmentation greatly improved the working efficiency in contouring the OARs (*P*<0.05).

## Discussion

The results of this study are relatively consistent with those of the challenge report of automatic segmentation of thoracic organs organized by the American Association of Physicists in Medicine (AAPM)’s annual meeting in 2017 (21),with the right lung having the highest average DSC (0.96) and the esophagus having the lowest average DSC (0.67). Compared with U-Net, the autosegmentation results of the OARs based on the proposed model were better with higher DSC as well as lower HD95 and ASD. Among them, DSC differences of the spinal cord and esophagus, HD95 differences of the spinal cord, heart, trachea and esophagus, as well as ASD difference of the spinal cord were statistically significant (P<0.05).

Lustberg T et al. (23) used a deep learning autosegmentation software (Mirada) to create thoracic OARs contours and the model was built by using 450 lung patients’ images as the training set. For 20 CT scans of stage I-III NSCLC patients in the testing set, the median DSCs of the spinal cord, the lungs, and heart were 0.83, >0.95, >0.90, respectively. Zhang T et al. (24) developed a 2D AS-CNN based on the ResNet101 network using a training dataset of 200 lung cancer patients. The average DSCs of the left lung, right lung, heart, spinal cord, and esophagus of 19 NSCLC patients were 0.94, 0.94, 0.89, 0.82, and 0.73, respectively. Zhu JH et al. (25) proposed an automatic segmentation model based on depth convolution to segment CT images from 36 lung cancer patients. The average DSCs of the lungs, heart, liver, spinal cord and esophagus were 0.95, 0.91, 0.89, 0.76 and 0.64, respectively. Dong X et al. proposed a U-Net-generative adversarial network (U-Net-GAN) and realized the segmentation of 5 thoracic OARs. Among them, the left lung, right lung, and heart were automatically segmented by a 2.5D GAN model, while the esophagus and spinal cord were automatically segmented by a 3D GAN model. The average DSCs of the left and right lungs, spinal cord, esophagus, and heart were 0.97,0.97, 0.90, 0.75, and o.87, respectively. He T et al. (26) proposed a uniform U-like encoder-decoder architecture abstracted from the U-Net and trained it using 40 patients’ thoracic CT scans. High DSC values were obtained for esophagus (0.86), heart (0.95), trachea(0.92) and aorta (0.95) from 20 patients in the testing set. Feng X et al. (27) developed a model based on 3D U-Net to autosegment thoracic OARs using 36 thoracic CT scans as the training set. The performance of the model was evaluated on two groups of testing set consisting of 12 patients and 30 patients, respectively. The average DSCs of the spinal cord, right lung, left lung, heart and esophagus of the first testing set reached 0.89, 0.97, 0.98, 0.93, and 0.73 while those of the second testing set were 0.85, 0.98, 0.98, 0.86 and 0.69, respectively.

The differences in all dosimetric metrics of the OARs between manual and automatic delineations were not statistically significant (P>0.05) in our study. The maximum dosimetric metrics differences were 0.41Gy for Dmean of the trachea and 0.64% for V5 of bilateral lungs, while the clinically acceptable dose difference and volume difference of OARs between manual and automatic delineation are supposed to be within 1Gy and 1%, respectively. Zhu J et al. (28) evaluated the performance of automatic segmentation of the OARs with dosimetric metrics for esophageal cancer patients. The maximum metrics differences were 0.35 Gy for Dmax of the spinal cord and 0.4% for V30 of bilateral lungs. The results in our study were consistent with those of the above study.

Due to the different training datasets, it is difficult to compare the advantages and disadvantages of the proposed model and the published model. However, the number of training cases used in our study was obviously fewer; the proposed model has strong feature extraction ability in the training of small samples, and the segmentation results are similar to those of the training model of relatively large datasets. A limitation of this study needs to be pointed out. That is, due to low soft tissue contrast, small volume, and large shape variability across patients, the automatic segmentation results of the esophagus are not ideal, and the DSC value is lower than 0.7, which is clinically unacceptable (29, 30), therefore, we did not take into account the esophagus when analyzing the dose-volume parameters in the treatment plan. In the next work, we need to further optimize the model and expand the size of data to increase its generalization and segmentation effect.

Currently, there are three main development directions for deep learning networks in medical image segmentation. The first direction is to deepen the network level and depth, extract deeper semantic features to obtain stronger expression ability, or widen the network to increase the number of channels to obtain more information in the same layer, such as the texture features of different grayscales and boundary features in different directions. The second direction is to obtain a more effective spatial feature extraction ability by learning the sequence concatenation properties of multiple CT slices of a patient, represented by 3D U-Net and many other derivative networks. The third direction represented by DenseNet is to improve the utilization rate of the feature map by sharing the feature map layer by layer to enhance the feature expression ability of the image and improve the generalization performance of the network (31).

## Conclusion

Compared with U-Net, the proposed model based on DenseNet is better in the OARs segmentation task; even if the training set has fewer images, it can still fairly effectively prevent the occurrence of overfitting. At the same time, it can effectively alleviate the problem of the gradient disappearing in the training process by repeatedly using different levels of feature maps, providing a new idea for medical image segmentation.

## Data Availability Statement

The original contributions presented in the study are included in the article/supplementary material. Further inquiries can be directed to the corresponding author.

## Ethics Statement

The studies involving human participants were reviewed and approved by the Ethics Committee of the Seventh Medical Center of Chinese PLA General Hospital. The patients/participants provided their written informed consent to participate in this study.

## Author Contributions

FZ and QW contributed conception and design of the study. FZ and AY trained the deep learning models. FZ and QW performed data analysis and drafted the manuscript. NL, HJ, DC, and YW helped to collect the data and evaluate radiotherapy planning. YY designed radiotherapy planning. All authors contributed to the article and approved the submitted version.

## Funding

This work was supported by the Beijing Municipal Science and Technology Commission (No.Z181100001718011).

## Conflict of Interest

The authors declare that the research was conducted in the absence of any commercial or financial relationships that could be construed as a potential conflict of interest.

## Publisher’s Note

All claims expressed in this article are solely those of the authors and do not necessarily represent those of their affiliated organizations, or those of the publisher, the editors and the reviewers. Any product that may be evaluated in this article, or claim that may be made by its manufacturer, is not guaranteed or endorsed by the publisher.
